# Inclusion of Cross-Linked Elastin in Gelatin/PEG Hydrogels Favourably Influences Fibroblast Phenotype

**DOI:** 10.3390/polym12030670

**Published:** 2020-03-17

**Authors:** Ye Cao, Bae Hoon Lee, Scott Alexander Irvine, Yee Shan Wong, Havazelet Bianco Peled, Subramanian Venkatraman

**Affiliations:** 1 School of Materials Science and Engineering, Nanyang Technological University, 50 Nanyang Avenue, Singapore 639798, Singapore; ycao@ntu.edu.sg (Y.C.); LBH217@HOTMAIL.COM (B.H.L.); SAIRVINE@ntu.edu.sg (S.A.I.); YSWONG@ntu.edu.sg (Y.S.W.); 2 The Inter-Departmental Program for Biotechnology, Technion-Israel Institute of Technology, Haifa 32000, Israel; 3 Department of Chemical Engineering, Technion-Israel Institute of Technology, Haifa 32000, Israel; 4 Subramanian Venkatraman, Materials Science and Engineering, National University of Singapore, Singapore 119077, Singapore

**Keywords:** polyethylene glycol hydrogel, myofibroblast, gelatin, elastin, cell encapsulation, dermal substitu

## Abstract

The capacity of a biomaterial to innately modulate cell behavior while meeting the mechanical property requirements of the implant is a much sought-after goal within bioengineering. Here we covalently incorporate soluble elastin into a gelatin–poly (ethylene glycol) (PEG) hydrogel for three-dimensional (3D) cell encapsulation to achieve these properties. The inclusion of elastin into a previously optimized gelatin–PEG hydrogel was then evaluated for effects on entrapped fibroblasts, with the aim to assess the hydrogel as an extracellular matrix (ECM)-mimicking 3D microenvironment for cellular guidance. Soluble elastin was incorporated both physically and covalently into novel gelatin/elastin hybrid PEG hydrogels with the aim to harness the cellular interactivity and mechanical tunability of both elastin and gelatin. This design allowed us to assess the benefits of elastin-containing hydrogels in guiding fibroblast activity for evaluation as a potential dermal replacement. It was found that a gelatin–PEG hydrogel with covalently conjugated elastin, supported neonatal fibroblast viability, promoted their proliferation from 7.3% to 13.5% and guided their behavior. The expression of collagen alpha-1(COL1A1) and elastin in gelatin/elastin hybrid gels increased 16-fold and 6-fold compared to control sample at day 9, respectively. Moreover, cells can be loaded into the hydrogel precursor solution, deposited, and the matrix cross-linked without affecting the incorporated cells adversely, thus enabling a potential injectable system for dermal wound healing.

## 1. Introduction

Two important considerations in the design of cell bearing scaffolds are the ability of the scaffold material to influence the functionality of the entrapped cells and secondly, to meet the mechanical property requirements for the scaffold [1,2]. Cell responses can be guided by cues incorporated into the scaffold. These cues can be chemical, biological, and physical in nature [3]. They mediate effects such as cellular attachment, migration, spreading, proliferation, phenotype, and extracellular matrix (ECM) remodeling [4,5]. Within natural ECM, certain motifs play a crucial instructive role in coercing cell behavior and function and thus such components can be exploited for biomimetic design and fabrication of scaffold biomaterials [1,6,7,8].

To date, the majority of hydrogels for cell encapsulation/tissue engineering are limited to a single ECM component or peptide type with poor mechanical properties, which eventually results in less than optimal regeneration of new tissue [9,10]. Hence, such hydrogels may have limited ability to guide cellular actions compared to more complex ECM protein combinations in vivo Biomimetic hybrid hydrogels have been developed to overcome these drawbacks: these hybrids combine the benefits of synthetic polymers, (tunable mechanical characteristics) and natural ECM proteins (providing biological cues) [11,12,13,14,15,16,17]. For example, bioinert poly (ethylene glycol) (PEG) hydrogels can be tailored with adhesive peptides or ECM proteins that can promote cell attachment, proliferation, and ECM deposition [18,19,20]. In our own previous study [21], gelatin was conjugated with one acrylate of poly (ethylene glycol) diacrylate (PEGDA) and then fibroblasts were encapsulated in the gelatin–PEG hydrogel by UV photopolymerization in situ. We found that the attachment and viability of fibroblasts in PEG hydrogels were improved with the inclusion of gelatin at a minimum concentration of ~2.3% (wt/v). Although gelatin–PEG hydrogels showed high cell viability, some of the cells maintained round shapes for several days. What’s more, the phenotype or function of encapsulated fibroblasts is not well guided as this biomimetic hydrogel only contain one biological cue. As we all know, the natural ECM is a complex, heterogeneous network of various proteins, growth factors and polysaccharides. Thus, it is critical to better mimic various features in the ECM in order to modulate cells innately.

In this paper, we further optimized above gelatin–PEG hydrogels by covalent incorporation of elastin in order to innately regulate cell fate and behavior in a cellularized skin substitute. Our approach here has been to look at the elastin effect (based on two different concentrations) on cell behavior while keeping the mechanical properties comparable. We hypothesize that inclusion of elastin into pre-optimized gelatin hybrid PEG gels can mimic ECM functionality more closely than collagen/gelatin natural gels (poor mechanical property overtime). In the case of skin regeneration, collagen-based scaffolds currently dominate with products such as Integra (Integra Life Science Corp.) being one of the most widely used commercially. Integra consists of a porous dermal layer fabricated from bovine collagen, chondroitin-6-sulfate, and a temporary silicone layer. Unfortunately, it also suffers from severe wound contraction and excessive scar formation [22,23]. It has been proven that the inclusion of elastin to the dermal substitutes is particularly important in dermal substitutes, as it is a key biological component to impart elastic properties into tissues and suppress adverse reactions during wound healing [24]. It also promotes a number of cellular responses including chemotaxis, proliferation, attachment, and differentiation [22,25]. Such properties have, to date, been under-utilized in the development of dermal substitutes, primarily due to the lack of a suitable and stable incorporation method. Despite years of research, there is no cytocompatible cross-linking approach linking elastin to the gelatin/collagen networks for the regeneration of dermal substitutes.

Previous studies utilizing elastin have primarily focused on incorporation methods (chemical conjugation or physical blending) [26,27,28] to fabricate two-dimensional (2D) cell attachment surfaces rather than producing cell bearing 3D hydrogels [29,30]. In these cases, cytocompatible cross-linking is not a priority. For example, soluble bovine elastin has been used to fabricate highly porous elastic hydrogels using cyto-damaging glutaraldehyde cross-linking and high pressure CO_2_ [31,32]. Such 2D studies have demonstrated the advantages of including solubilized elastin for cell attachment and proliferation [33], but do not replicate the 3D microenvironment of ECM [15]. Recently, a natural elastin-based, thermo-responsive injectable hydrogel, fabricated by cross-linking alpha-elastin, poly (N-isopropylacrylamide), polylactide, 2-hydroxyethyl methacrylate and oligo (ethylene glycol) has been assessed for the encapsulation of fibroblasts. The system, however, was not optimal since the encapsulated fibroblasts within the hydrogel underwent a 20% loss of viability. The effects of elastin on cellular morphology, phenotype and the extent of ECM remolding was not characterized for this hydrogel [34]. The reasons for the unsatisfactory cell behavior in this elastin-based thermoresponsive hydrogel may be factors such as the suboptimal presence of cell attachment sites (e.g., the arginylglycylaspartic acid (RGD)) [35], as well as inappropriate mechanical characteristics [9,21]. Thus, the effects of the incorporation of elastin inside hydrogels, on cell behavior have yet to be fully elucidated for tissue engineering applications, particularly in a 3D microenvironment.

In this study, we hypothesize that inclusion of the covalently bound, initially soluble elastin in the form of elastin–PEG–acrylate into a gelatin–PEG hydrogel will act as an instructive cue, guiding the phenotypic behavior of encapsulated Normal Human Dermal Fibroblasts (NHDFs).In particular, such hybrid gels cannot only be more easily fine-tuned with respect to their mechanical properties, but also promote the desired cell fate while facilitating the delivery of encapsulated cells to the injured skin site. In addition, elastin–PEG–acrylate and gelatin–PEG–acrylate amounts in the hybrid gel can be adjusted according to the cell types and applications. We demonstrate that an elastin/gelatin PEG hydrogel can incorporate viable cells and undergo cytocompatible gelation. The modification of the gelatin–PEG hydrogel with elastin incorporation was assessed for its effect on the following: (a) fibroblast proliferation; (b) ECM protein deposition (collagen and elastin), and (c) differentiation towards myofibroblasts. This included an assessment of cellular morphology, proliferation, F-actin expression, and ECM remolding within the 3D hydrogel culture systems. The outcome of this study was the development of a multi ECM-protein-incorporated PEG hydrogel with tunable mechanical properties, and incorporating cell guiding cues with potential application towards dermal bioengineering.

## 2. Materials and Methods

### 2.1. Gelatin and Elastin PEGylation

**Figure 1:**
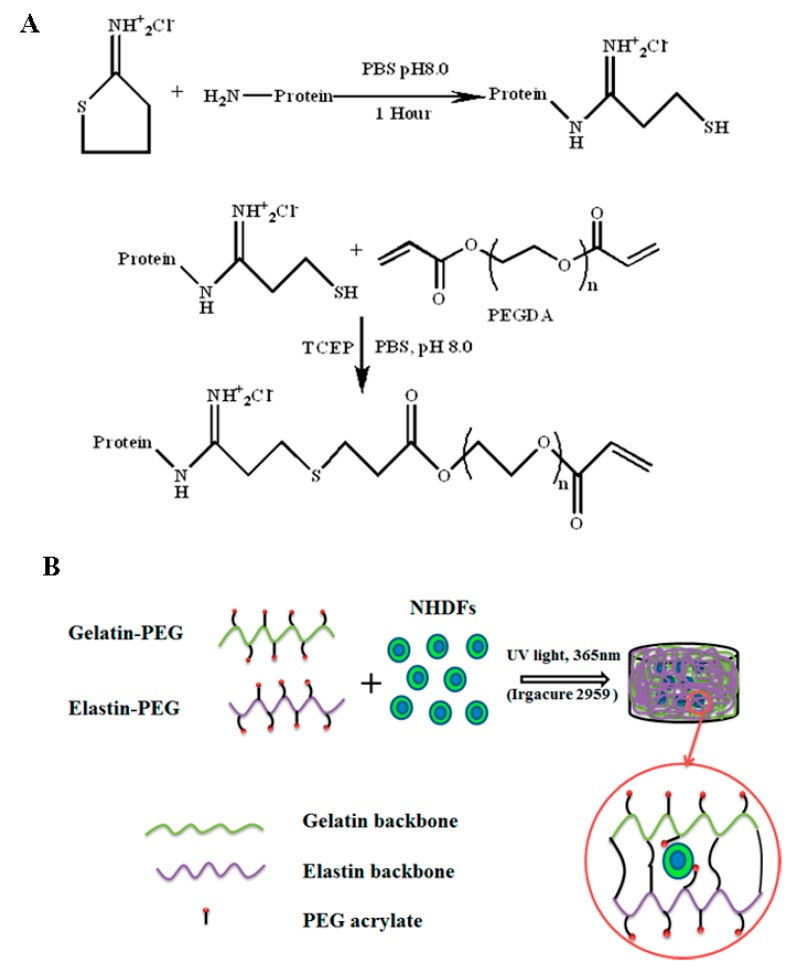
(**A**) Synthesis of gelatin–Polyethylene glycol-acrylate (Gelatin-PEG) and elastin– Polyethylene glycol-acrylate (elastin-PEG) precursor. Gelatin and elastin were modified with free sulfhydryl group on the backbone via reacting with Traut’s Reagent. (**B**) Cell encapsulation in 3D hydrogel via UV photopolymerization. (NHDFs: Neonatal human dermal fibroblasts).

Poly(ethylene glycol) diacrylate (PEGDA) was synthesized from linear 8 kDa molecular weight PEG-OH (Sigma-Aldrich Corporation, Singapore) as previously described [36]. The thiolation of gelatin (type A, 175 bloom, average molecular weight 40 kDa from porcine skin, Sigma-Aldrich Corporation) was performed according to our previous published paper [21] (Figure 1). Sulfhydryl groups were conjugated to gelatin by reacting the primary amine group of gelatin with Traut’s Reagent (Pierce Thermo Fisher, Singapore). This served as a precursor to synthesize gelatin–PEG (GP, Figure 1A). 2.40 g (0.00060 mol) of gelatin was dissolved in 240 mL of pH 8.0 phosphate-buffered saline (PBS) (0.1mol/L) solution and then 120 mg (0.87 mmol) of Traut’s Reagent was added to the above solution to react for 1 h at 37 °C. The unreacted Traut’s Reagent was removed by Tangential flow filtration (TFF, Pall life sciences, New York, USA) with 30 kDa molecular weight cutoff (MWCO) capsule at 37 °C, while pH 3.5 distilled water prepared by adding hydrogen chloride (Sigma-Aldrich, Singapore) was used as a purification solution. The amount of sulfhydryl after thiolation was measured by Ellman’s reagent (Sigma-Aldrich Corporation, Singapore), while one fold molar amount of Tris(2-carboxyethyl) phosphine hydrochloride (TCEP, Thermo Fisher, Singapore) was added to the above solutions to improve the subsequent Michael-type addition reaction as TCEP can inhibit the formation of a disulfide bond [37]. The pH of solutions was adjusted to pH 8, while a 4-fold molar excess of PEGDA dissolved in pH 7.4 PBS (0.1 mol/L) was added to the solutions and reacted overnight at 37 °C. The final product was purified and concentrated by MWCO 70 kDa TFF capsule in Dulbecco’s phosphate buffered saline (DPBS, Lonza, Singapore), filtered by 0.22 µm filter in a biosafety hood and stored in –80 °C. Soluble elastin of molecular weight ranging from 3000–60000, from bovine neck ligament (American Elastin Product Company, Missouri, USA) was used to synthesize the elastin–PEG precursor by the same method and reaction ratio, while the purification was finished by 10 kDa (after thiolation) and 50 kDa (after PEGylation) MWCO TFF capsule, respectively. The final product was lyophilized and stored at 4 °C. The gelatin and elastin amount in the precursor was determined using the BCA kit assay (Pierce Thermo Fisher, Singapore). The amount of PEG was calculated by deducting the gelatin or elastin and PBS salts amount from the total weight of lyophilized powder. BCA standard curves for gelatin and elastin at concentrations of 1500, 1000, 500, 250, 125, 62.5 mg/mL (were prepared, giving regressions of Y= 0.0002X+0.0166 (R^2^ = 0.999) and Y = 0.2217X (R^2^ = 0.998). The Proton nuclear magnetic resonance (^1^H NMR) spectra were recorded with a 400 MHz spectrum (Bruker, NanoBay 400MHz, Singapore) using deuterium oxide.

### 2.2. Gelatin and Elastin Hybrid PEG Hydrogel Preparation

Gelatin–PEG and Elastin–PEG hybrid hydrogels were covalently cross-linked using photo-polymerization by adding 0.1 w/v % Irgacure 2959 (Sigma-Aldrich Corporation), exposing to UV light (365 nm, 1–1.5 mW/cm^2^, Viber, German) for 5 min. After thaw gelatin–PEG at 37 °C, Elastin–PEG precursor was dissolved in gelatin–PEG precursor. The hydrogels were defined as “GEPX” or “GPE control” and shown in Table 1, where “GEP” represents covalently cross-linked (gelatin–PEG and elastin–PEG hybrid hydrogel. “X” is the concentration of elastin–PEG precursor (%(wt/v)), while the concentration of Gelatin–PEG is fixed at 48 mg/mL (4.80% (wt/v)). Different amounts of elastin–PEG–acrylate were then mixed with a constant quantity of gelatin–PEG–acrylate to investigate how cell behavior could be guided by elastin content. Since the main hypothesis of this work was that elastin in a 3D scaffold would influence cell behavior favorably, we wanted to isolate the effect. So the pre-optimization was to ensure two things: (1) The scaffolds are mechanically equivalent (starting moduli similar); (2) The elastin content varies across the samples. “GPE control” represents the gel fabricated from gelatin–PEG precursor 48 mg/mL (4.80% (wt/v)) with 9 mg/mL (0.90% (wt/v)) PEGDA and 15 mg/mL (1.50% (wt/v)) physically incorporated soluble elastin. The control was designed to compare covalently-bound elastin with physically-incorporated soluble elastin on their effect on fibroblasts, which would be helping to demonstrate that chemical anchoring of the elastin is necessary. The 0.90% (wt/v) PEGDA was added to the control in order to obtain comparable mechanical properties as acrylate groups in elastin–PEG (GEP45 and GEP30) also could increase the mechanical properties.

### 2.3. Characterization of Hydrogels

Gelatin/ elastin–PEG hydrogels were formed from 100 µL of precursors, washed in distilled H_2_O for 24 h and then lyophilized for 48 h. Swelling studies were performed by immersing the lyophilized hydrogels in 2.0 mL of pH 7.2 PBS and incubating at 37 °C in 24-well plates. At predetermined time points, the hydrogels were weighed. The swelling ratio (Q) is calculated as Q = (W_2_−W_1_)/W_1_, where W_2_ is the gel weight at each time point and W_1_ is the original dried weight of the hydrogel. Following each essay, the swelling medium was replenished.

To determine the possible effect of collagenase on hydrogel degradation, each hydrogel was prepared from 100 µL precursor solution as described above, placed in 24-well plates with 1 mL DPBS containing 0.1 mg/mL or 0.5 mg/mL collagenase type I A [25]. At each time point, the weight of the remaining gel (W_3_) was measured and immersed in fresh collagenase solution until the hydrogel was completely degraded. The percentage of weight loss was calculated as (W_4_−W_3_/W_4_) × 100%, where W_4_ represents the hydrogel weight after the initial 12 h DPBS equilibration.

The gel fraction of the cross-linked hydrogels was determined by the method described by Gobril et al. [38]. Each hydrogel fabricated from 100 µL precursor was immersed in 2 mL distilled H_2_O at room temperature for 24 h and lyophilized for 48 h. The residue after extraction was taken as the gel component. The gel fraction of the cross-linked hydrogels was calculated as (W_3_/W_4_) × 100%, where W_3_ and W_4_ are weights of lyophilized hydrogels after soaking in deionized water and 100 µL hydrogel precursor sample, respectively.

The rheological characterization was performed using an Anton Paar Physica MCR 501 rheometer equipped with Peltier plate temperature-controlled systems (P-PTD-200/TG and P-PTD120/GL). A CP25 cone-plate geometry with a 25 mm diameter was used for shear viscosity experiment. For cell-free and cell encapsulated hydrogels, a PP8 parallel-plate of 8 mm diameter was used to measure the storage modulus (G’) of each gel at day 0 (d0), d1, d3, and d7. The testing conditions for all measurements were 1% strain amplitude at an oscillation frequency of 0.1-4 Hz, which is in the linear viscoleastin region.

The degree of functionalization of gelatin or elastin with PEGDA was quantified as previously described via the trinitrobenzenesulfonic acid assay (TNBSA, Sigma-Aldrich Corporation, Singapore) [39,40]. The conjugation efficiency was calculated using equation ($1-\frac{Amine\;amount\;after\;gelatin\;conjugation}{Amine\;amount\;before\;gelatin\;conjugation})\times 100\%$. The final network cross-linking density ρ was calculated from Flory and Rehner theory where *ρ_x_* = *ρ_PEGDA_*/$\bar{M_{c}}$ (*ρ_PEGDA:_* the density of PEGDA, $\bar{M_{c}}$: the average molecular weight between corrslinks [41]. The initial average mesh size and *M_c_* was calculated using Equations (1)–(6) according to previously published papers [21,42,43].

(1) $$Q_{m}=\frac{M_{s}}{M_{d}}$$

(2) $$Q_{V}=1+\frac{\rho_{PEGDA}}{\rho_{Water}}\left(Q_{m}-1\right)$$

(3) $$V_{2,s}=\frac{1}{Q_{v}}$$

(4) $$G^{\prime }=0.5\times \left(\frac{dRT}{M_{c}}\right)C_{x}$$

(5) $${\left({\bar{r}}_{0}^{2}\right)}^{1/2}=l{\left(C_{n}\frac{2\bar{M_{c}}}{{\bar{M}}_{r}}\right)}^{1/2}$$

where *Q_m_* is the initial mass swelling ratio, *Q_v_* is the initial volume swelling, *M_s_* is the swollen mass of the gel, *M_d_* is the dry gel mass, *ρ_PEGDA_* is the PEGDA density (1.21 × 10^3^ kg/m^3^), *ρ_water_* is the water density, V_2,s_ is the polymer volume fraction, $\chi_{1\;}$is the solvent-polymer interaction parameter (0.426), G’ is the shear storage modulus of gels, $\bar{r_{0}^{2}}$ is the root-mean-square distance between cross-links, M_r_ is the molar mass of the repeating unit (44 g/mol for PEG), l is the C-C bond length (1.54 × 10^−10^ m), and $C_{n}$ is the characteristic ratio (4.0 for PEG) [44].

### 2.4. Cell Encapsulation in Gelatin–PEG Hydrogel

NHDFs were purchased from Lonza Bioscience company and cultured in fibroblast basic medium-2 (FBM-2) with a FGM-2 SingleQuot Kit supplement (Lonza Bioscience Company, Singapore). Cell (passage 4-7)-seeded gelatin–PEG and elastin hybrid hydrogels were prepared. The precursor solution was made by dissolving the elastin–PEG powder into gelatin–PEG solution with 0.1 w/v % Irgacure 2959 at 37 °C as the gelatin–PEG precursor was purified and stored in PBS (mentioned in Section 2.1) at –80 °C. Cell-seeded constructs were made from 100 μL aliquots of the cells in a suspension of gelatin–PEG and elastin–PEG to give a final cell density of 2 × 10^6^ cells/mL (Figure 1B). The cell bearing solution was deposited into a flat-bottom 96-well plate as the mold. After UV photopolymerization, the cell-seeded hydrogels were transferred into ultra-low cell attachment 6-well plates (Corning, New York, NY, USA), washed with PBS and immersed in the culture medium. Cell encapsulated elastin–PEG only hydrogel (45 mg/mL) was prepared by the same method as gelatin/elastin hybrid PEG hydrogels.

### 2.5. Cell Proliferation

The proliferation of NHDF was determined using Click-It 488 EdU flow cytometry assay kit (Invitrogen, Carlsbad, CA, USA). Cell encapsulated hydrogels were immersed in 10 µM EdU (5-ethynyl-2′-deoxyuridine) in culture medium and incubated for 24 h (normally 1–2 h incubation for 2D culture) as 3D encapsulated cells proliferate much slower than 2D cultured cells. At day 1, 3, 7 and 9, the NHDFs in hydrogels (cross-linked from 100 µL precursor) were harvested by degrading the gels in 2 mg/mL collagenase type I A (Sigma) for 2h at 37 °C. Subsequently, the cells were collected by centrifugation, washed twice with DPBS, fixed and stained according to the assay kit protocol. The percentage of proliferation cells was measured by using a flow cytometer (LSR-II, Becton Dickinson, NJ, USA).

### 2.6. Cell Live/Dead and Cell Morphology

NHDFs encapsulated in gelatin–PEG hydrogels were stained with live/dead stain (2 mM Calcein-AM/4 mM EthD-1, Invitrogen, California, USA) and imaged by a fluorescence microscope (Olympus, CX 51, Tokyo, Japan).

### 2.7. Immunofluorescence Staining of ECM Protein Deposition

On day 9, the hydrogels containing NHDFs were washed three times in DPBS and fixed in 3.7% paraformaldehyde (PFA) in DPBS for 30 min. Subsequently, the hydrogels were immersed in 0.1% Triton X-100 in DPBS for 30 min at room temperature to permeabilize the cell membranes. For collagen type I and elastin staining, the hydrogels were blocked in 10% horse serum in DPBS for 1 h. The monoclonal mouse anti-collagen type I antibody (Abcam, Cambridge, UK) at 1/500 dilution in 10% horse serum was added to the hydrogels and the samples were incubated at 4 °C for 12 h. The hydrogels underwent 3×10 min washes in DPBS before incubation in 1/200 dilution of Alexa Fluor 555 Goat Anti-Mouse IgG Secondary Antibody (Abcam, Cambridge, UK) in 10% horse serum in DPBS for 3 h at room temperature. A similar procedure was used to stain elastin, using a primary monoclonal rabbit anti-human elastin antibody (Abcam, Cambridge, UK) and Alexa Fluor 488 conjugated goat anti-rabbit secondary antibody (Abcam, Cambridge, UK). For F-actin cytoskeleton staining, after 3 washes in DPBS and hydrogels were soaked in a solution with Alexa Fluor 568 phalloidin at 5 IU/mL (Invitrogen, Cambridge, USA) for 90 min incubation at room temperature. Cellular nuclei were stained with 300 nM 4′,6-diamidino-2-phenylindole (DAPI) (Invitrogen, Califonia, USA). Confocal imaging was performed using a CLSM, Leica SP2 inverted microscope (Wetzlar, Germany). The immunofluorescence staining images of cell-free hydrogels were shown in supporting information.

### 2.8. Gene Expression of Encapsulated Cells

The relative ECM protein (collagen IA and elastin) gene expression was measured by reverse transcription followed by quantitative real-time polymerase chain reaction (RTPCR). NHDFs in cell-laden hydrogels (prepared from 150 µL precursor) were harvested from the gel at day 9 by degradation with 2 mL of collagenase type I A in DPBS (2mg/mL) for 2 h at 37 °C. The released cells were collected by centrifugation and washed twice with DPBS. Total RNA was extracted using a Taq Probe qPCR Mastermix (Sangon Biotech, Shanghai, China) according to the assay kit protocol. The cDNA was prepared using an iScript™ cDNA Synthesis Kit (Bio-Rad Laboratories, California, USA) and a thermal cycler (Eppendorf, Hamburg, Germany). A KAPA SYBR^®^ FAST qPCR Kit (Biosystems, Basel, Switzerland) was used with the CFX 96 Real-Time PCR System (Bio-Rad Laboratories, California, USA) for doing Real-time PCR in duplicate for each sample, while glyceraldehyde-3-phosphate dehydrogenase (GAPDH) was employed as an internal control. Elastin (ELN) Primers used were 5′-GGCCATTCCTGGTGGAGTTCC-3′ as the forward primer and 5′-AACTGGCTTAAGAGGTTTGCCTCCA-3′ for the reverse primer. Collagen alpha-1 (COL1A1) Primers used were 5′-GAATTCCAGCTGTCTTATGGCTATG-3′ as its forward primer and 5′-AGATCTAGCCCGGTAGTAGCG-3′ for the reverse primer. Forward primers of alpha-SMA were 5′-GACAGCTACGTGGGTGACGAA-3′, while the reverse primers were 5′-TTTTCCATGTCGTCCCAGTTG-3′. was designed like the forward primer and reverse primer of GAPDH were 5′-TGCACCACCAACTGCTTAGC-3′ and 5′-GGCATGGACTGTGGTCATGAG-3′, respectively [45]. Gene expression results were normalized to the internal gene, GAPDH, using the 2^−(∆∆Ct)^ [28].

### 2.9. Statistical Analysis

Data are presented as mean ± standard deviation of samples in at least three experiments and analyzed by ANOVA. A value of *p* < 0.05 was considered statistically significant.

## 3. Results and Discussion

### 3.1. Characterization of Gelatin–PEG and Elastin–PEG Modification

**Figure 2:**
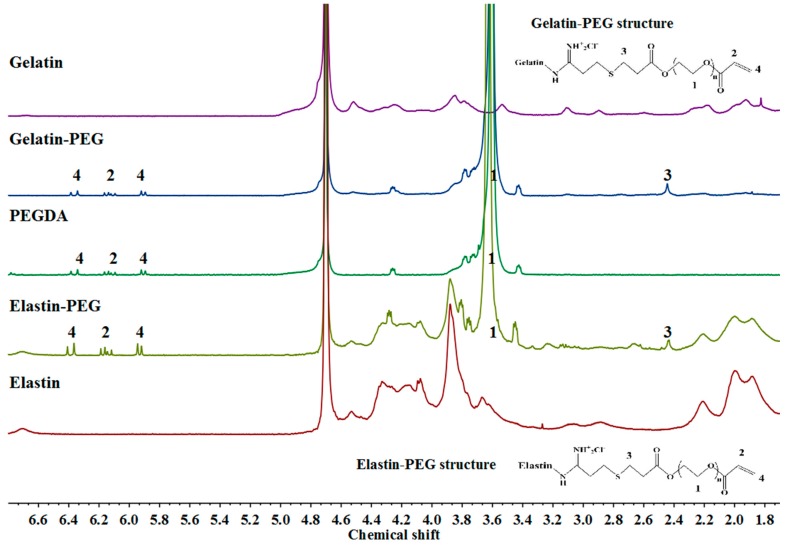
^1^H Nuclear magnetic resonance (NMR) spectra of gelatin, elastin, PEGDA, gelatin–PEG–acrylate, and elastin–PEG–acrylate.

The conjugation of gelatin–PEG–acrylate and elastin–PEG–acrylate was confirmed by ^1^H Nuclear magnetic resonance (NMR) spectroscopy (Figure 2). PEGDA exhibited three chemical shift peaks (protons in acrylates) at peak 1 (3.6 ppm, -OCH_2_CH_2_-), peak 2 (6.1 ppm, -CH=CH_2_), and peak 4 (5.8 and 6.4 ppm, -CH=CH_2_) in Figure 2,while both gelatin–PEG–acrylate and elastin–PEG–acrylate have a new peak 3 (2.4 ppm) from the methylene protons present on the Traut’s Reagent moieties [36,46]. After PEG conjugation, the spectrum of gelatin–PEG–acrylate and elastin–PEG–acrylate showed a new peak of sulfhydryl groups and also the similarity of both original proteins and PEGDA. So the NMR spectrums confirm the sulfhydryl groups and PEGDA were successfully conjugated to gelatin and elastin, respectively. The constitution of gelatin–PEG–acrylate and elastin–PEG–acrylate precursor are shown in Table 2. The protein concentration in both precursors was approximately 60% according to BCA assay results (Table 2).

### 3.2. Hydrogel Swelling, Degradation, and Mechanical Properties

The bulk properties of the hydrogel, such as swelling ratio and elastic modulus, have a direct impact on cell behavior. Furthermore, the architecture of the hydrogel is directly related to its porosity and swelling, both of which affect the diffusion of oxygen and nutrients to the cells. Three hydrogels were selected for study mechanical properties: GEP45 (covalently cross-linked gelatin–PEG and elastin–PEG hybrid hydrogel with 4.50 wt./v % elastin–PEG–acrylate), GEP30 (covalently cross-linked gelatin–PEG and elastin–PEG hybrid hydrogel with 3.00 wt./v % elastin–PEG–acrylate) and GPE control (gelatin–PEG–acrylate with physically-incorporated soluble elastin). The composition and preparation method of these hydrogels are detailed in Section 2.3.

The gel fractions of the resultant gelatin and elastin hybrid PEG hydrogels were investigated to evaluate the efficiency of the cross-linking process. The gel fractions were 80.4% ± 9.5%, 78.8% ± 12.4%, 65.8% ± 11.6% for GEP45, GEP30 and GPE control, respectively (*P* < 0.05). The results of the gel fraction study indicate that the GPE control has the lowest gel fraction since the physically incorporated elastin leached following immersion in distilled water for 24 h. The gel fractions of GEP45 and GEP30 were found to be similar to other UV photo-cross-linked PEG hydrogels [47].

**Figure 3:**
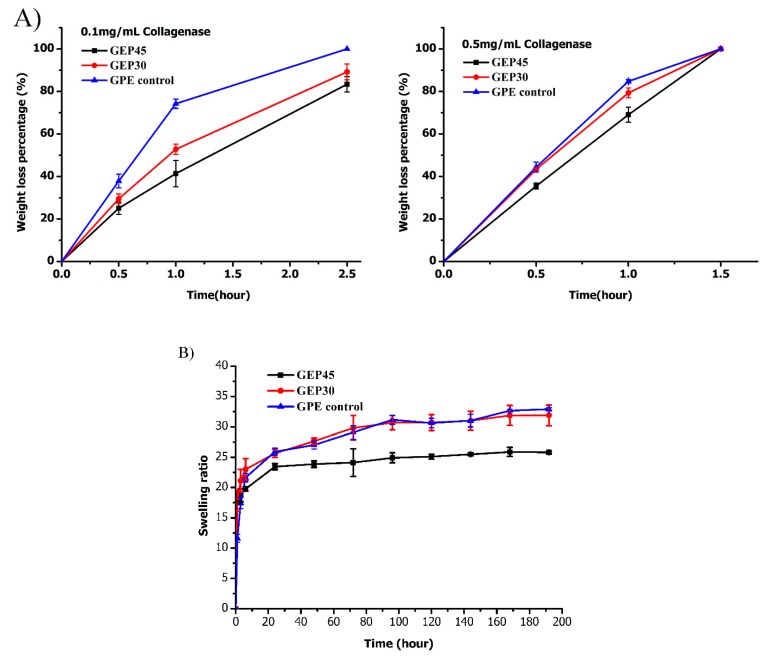
(**A**) Degradation kinetics of GEP45, GEP30 and GPE control in 0.1 mg/mL and 0.5 mg/mL collagenase solution. (n = 3); (**B**) Swelling profiles of GEP45, GEP30 and GPE control (n = 3).

Proteolytic degradation is a basic feature for a cell-encapsulated scaffold that allows cells to rebuild their surrounding microenvironments in situ. Gelatin and elastin-based materials are often applied in tissue engineering, based partly on their proteolytic susceptibility and partly on the ease with which the incorporated cells can remodel the ECM. Accordingly, the biodegradation kinetics of GEP45, GEP30 and GPE control in 0.1 mg/mL and 0.5 mg/mL collagenase type I A solution was studied and shown in Figure 3A. Collagenase specifically recognizes and hydrolyzes the X-Gly peptide bond of the peptide sequence Pro-X-Gly-Pro (X: neutral amino acids), since collagen and gelatin contain this sequence [48,49]. All hydrogels fully degraded after 3h (0.1 mg/mL collagenase I A) or 1.5h (0.5 mg/mL collagenase I A) incubation. The GEP45 and GEP30 hydrogels degraded slower than GPE control because of covalently cross-linking elastin–PEG; the hydrogel with larger elastin–PEG content displays a slower degradation rate (GEP30>GEP45). Both elastin and PEG cannot be degraded by collagenase. Different amounts of elastin–PEG–acrylate in GEP45 and GEP30 or extra PEGDA in GPE control caused different cross-linking density and affected the degradation speed although all the gels have the same gelatin–PEG–acrylate amount. Hydrogels incubated with 0.5 mg/mL collagenase displayed larger mass loss than those immersed in 0.1 mg/mL collagenase at the same incubation time. Thus, the PEGylation of gelatin and elastin did not obstruct the proteolytic degradation of gelatin. It should be borne in mind that such high concentrations of collagenase are not realized in vivo, and hence it is expected that in vivo biodegradation rates would be slower.

For cell studies, fibroblasts are incorporated into the hydrogel when it was in the nonequilibrium swollen state. Subsequently, when exposed to the excess cell culture medium, the hydrogel swells until an equilibrium state is reached. To understand the change of hydrogel swelling properties during the experiment period, the swelling characteristics of the gelatin and elastin hybrid PEG hydrogels were studied and are summarized in Figure 3B. Both the covalently cross-linked gelatin/elastin hybrid hydrogels and the physically incorporated elastin hydrogel (GPE control) displayed rapid water absorption and swelling to near maximum swelling ratio within 1–2 days (Figure 3B). GEP45 displayed the lowest mass swelling ratio as it was formed by a greater concentration of cross-linkable polymer (elastin + gelatin)-PEG. Conversely, the swelling of GEP30 hydrogels resulted in looser and more permeable polymer networks. GPE control has the lowest concentration of cross-linkable polymer and therefore displays a similar swelling ratio as GEP30. There was no statistically significant difference between GEP30 and GPE control (*p* > 0.05), while GEP45 had statistical difference with GEP30 and GPE control (*p* < 0.05). It is feasible, therefore, to tune the cross-linking density by altering the precursor amount (acrylate amount), with an inverse effect on the swelling ratio.

**Figure 4:**
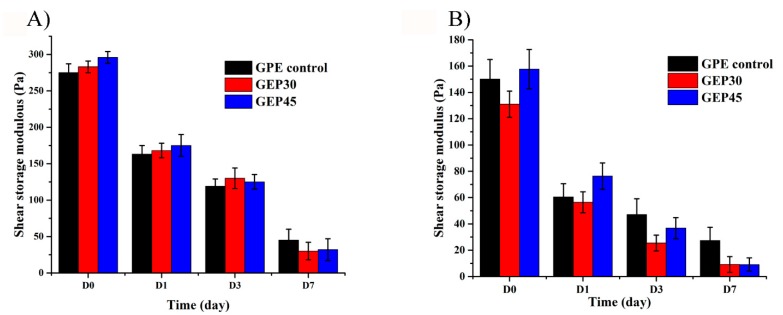
(**A**) The storage moduli changes of cell-free gelatin and elastin hybrid PEG hydrogel over 7 days (n = 3); (**B**) The in situ storage modulus change of various cell encapsulated gelatin and elastin hybrid PEG hydrogel over 7 days (n = 3).

Spatial confinement of cells restricts cytoplasmic spreading and cellular movement which can interfere with functionality [9]. It is generally accepted that storage modulus has an inverse relationship with the porosity (mesh size of the network) available to cells [50]. For example, poly (vinyl alcohol)-based copolymer hydrogels with the G’ in the range of 0.30–2.5 kPa have been used to encapsulate stem cells, while the proliferation significantly depended on the storage modulus of the hydrogels. They found that the cells would not proliferate when the storage modulus of the hydrogels was more than 1.1 kPa [51]. Our previous study proved that suitable initial storage modulus accelerates cell attachment [21]. Therefore, the storage moduli of cell-free and cell encapsulated all three hydrogels were monitored over a time course by rheometer and shown in Figure 4A,B, respectively. For cell-free hydrogels, GEP45 showed the highest G’ of 296 ± 8 Pa, as it was fabricated with the highest concentration of cross-linkable polymer. The G’ of GEP30 and GPE control were similar at 283 ± 8 Pa and 275 ± 12 Pa, respectively (*P* > 0.05), although the difference between G’ of GEP45 and GPE control (*P* < 0.05), was statistically significant. GPE control is composed of gelatin–PEG–acrylate, PEGDA and free elastin, which is cross-linked from PEGDA and gelatin–PEG–acrylate with free elastin embedded. Extra PEGDA was added to have similar G’ and thus isolate the influence of elastin alone. Thus, GPE control would have similar G’ compared to GEP45 and GEP30. The cross-linking densities and initial mesh sizes calculated from initial G’ values (day 0) and Q (the theoretical dry mass multiply with gel fraction) are listed in Table 1. GEP45 displayed the highest cross-link density (6.34 × 10^−5^ ± 0.67 × 10^−5^ mol∙cm^−3,^), higher than the GEP30 hydrogels (4.04 × 10^−5^ ± 0.58 × 10^−5^ mol∙cm^−3^) while GPE control had the lowest cross-linking density (2.15 × 10^−5^ ± 0.17 × 10^−5^ mol∙cm^−3^). These results are in line with the swelling data, while the swelling increased with a decrease in the cross-linking density. The calculations for initial mesh sizes generated different values for all three hydrogels, with 46.4 ± 4.2 nm for the GPE control, 22.4 ± 3.5 nm for GEP45 and 30.3 ± 4.1 nm for GEP30 (*P* < 0.05). These results showed the same trend as the cross-linking density results. Since our approach was to keep the modulus constant (which depends on both cross-link density (mesh size) as well as solids content (per Equation (4)), our gels do differ in mesh size, but the starting moduli are similar.

The storage moduli of the hydrogels by day 1 decreased by approximately 130 Pa (~40%) for the cell-free samples, and by 80 Pa (~57%) for the cell-containing hydrogels. This drop is due primarily to swelling changes (Figure 3B). As the cell-laden gels start out with lower cross-link density, the percentage drop-in chain scission should be higher than that in cell-free samples. The G’ of GEP45, GEP30 and GPE control hydrogels showed a continuous decrease during the following 7 days. Since hydrogels are highly swollen and hydrophilic, degradation occurs through the bulk hydrogel. Gelatin/elastin hybrid hydrogels are fabricated through the cross-linking of PEG–acrylates, while each PEGDA molecule has two ester bonds. After swelling is reached 90% after 1 day, the G’ is still decreasing. The decrease in modulus following equilibrium swelling must be due to chain scission or de-cross-linking. Because the ester bonds in PEG-DA and Gelatin–PEGDA/elastin–PEGDA are more susceptible to hydrolysis [52]. In summary, the modulus decrease over time is due substantially to swelling (day 1), and decreases to very low values by day 7, with the cell-incorporated hydrogels always having lower moduli throughout.

The bulk properties of the hydrogels may also be influenced by the presence of encapsulated cells [53]. In Figure 4B, the in situ G’ of the three hydrogels (with incorporated cells) were measured using rheometry. All the cell encapsulated hydrogels have a similar initial storage modulus on day 0 (GEP45 = 157.7 ± 14.9 Pa, GEP30 = 131.0 ± 10.0 Pa, and GPE control = 150.0 ± 15.9 Pa, Figure 4B), although the differences between G’ of GEP45 and GEP30, as well as the G’ of GEP30 and GPE Control, were statistically significant.

The storage moduli of GEP45, GEP30 and GPE control hydrogels decreased rapidly after swelling over 24 h and then continuously decreased during the following 7 days (Figure 4B). Additionally, the initial G’ of cell encapsulated hydrogels was lower than that of the cell-free hydrogels cross-linked under the same conditions. The possible reason is that the high concentration of cells (2 million cells/mL precursor) decrease the cross-linking efficiency as the cells occupy a considerable volume (> 2000 µm^3^) that may separate the cross-linkable chains significantly.

The injectability of GEP45, GEP30, and GPE control was studied by measuring the complex viscosity at 1Hz are 44.0, 22 and 0.2 Pa∙s. The viscosities of GEP30, GEP45 and GPE control precursors were measured at three different shear rates of 0.1, 230 and 500 s^−1^ (Table 3). The viscosity of GEP30 and GEP45 decreased with increasing the shear rates as expected of shear-thinning materials. The shear-thinning property is a prerequisite for hydrogel injectability, hence the exerted shear stress at the nozzle site can be controlled, taking into account the syringe nozzle dimension and printing pressure [54].

### 3.3. 3D Cell Encapsulation and Cellular Behavior

Hydrogel scaffolds are considered a plausible skin substitute for grafting, as they have a 3D ECM like the constitution and mechanical properties, and can act as a template for regeneration [55]. The regeneration of badly damaged dermis, which is associated with deleterious contraction and scar formation, is a challenge for tissue engineering [55]. Severe wound contraction leads to excessive scar formation and distortion of the surrounding tissue, resulting in cosmetic disfigurements and joint immobility [56]. Hence, the targets for skin regeneration therapy are to decrease wound contraction, improve scar appearance and functionality, and aid wound healing [22]. It has been shown that the coating of a collagen dermal matrix with physically-incorporated elastin, reduced collagen contraction and improved tissue regeneration compared to a collagen-only matrix [57,58]. Daamen et al. demonstrated that, in vivo, lyophilized and carbodiimide cross-linked collagen/elastin scaffolds were able to promote both cell proliferation and further elastin deposition following subcutaneous implantation into rats [29]. In comparison, collagen-only scaffolds failed to promote elastin synthesis. Moreover, elastin has also been observed to suppress the accumulation of myofibroblasts that leads to wound contraction and scar tissue formation [59]. Therefore, elastin deserves consideration as an integral component of hydrogel dermal substitutes due to its fundamental structural and functional role in the skin.

In this study, the goal of this manuscript is to explore a skin-ECM mimicking cell-laden hydrogel. We would like to explore the possibility of elastin as an ECM biological cue (not mechanical property) for the regeneration of ECM. Our gelatin/elastin hydrogel was used to rebuild new skin ECM instead of providing mechanical support. Elastin used in this study is hydrolysis product of elastin fibers, which cannot provide elastic property to the hydrogel. In the following, we examine how the inclusion of elastin to gelatin–PEG hydrogel affected fibroblast proliferation, ECM protein deposition (collagen and elastin), F-actin expression, and differentiation towards myofibroblast within the 3D hydrogel culture systems.

In this study, NHDFs were encapsulated into elastin–PEG–acrylate only hydrogels, while cells were round shape until day 5 (supporting document Figure S2). We noticed that NHDF could not attach in 3D elastin only PEG hydrogel. Fibroblasts are anchorage-dependent cells, hence the degree of interaction with their scaffold has considerable implications for their functionality and viability [9,60]. Cells suspended in bioinert hydrogels such as PEG-based ones are unable to alter the morphology of their cytoplasm due to their lack of attachment with the surrounding environment. Such cells are reduced to discrete spherical morphology [9]. The interactions between fibroblasts and their 3D microenvironments are enabled through the binding of membrane-bound integrins to surrounding adhesive groups [9]. Inspired by natural ECM, gelatin is selected to promote NHDF attachment inside 3D gels because it is a potent cell-adhesive substrate and rich in cell-binding peptide sequences, such as the well-known RGD sequence.

**Figure 5:**
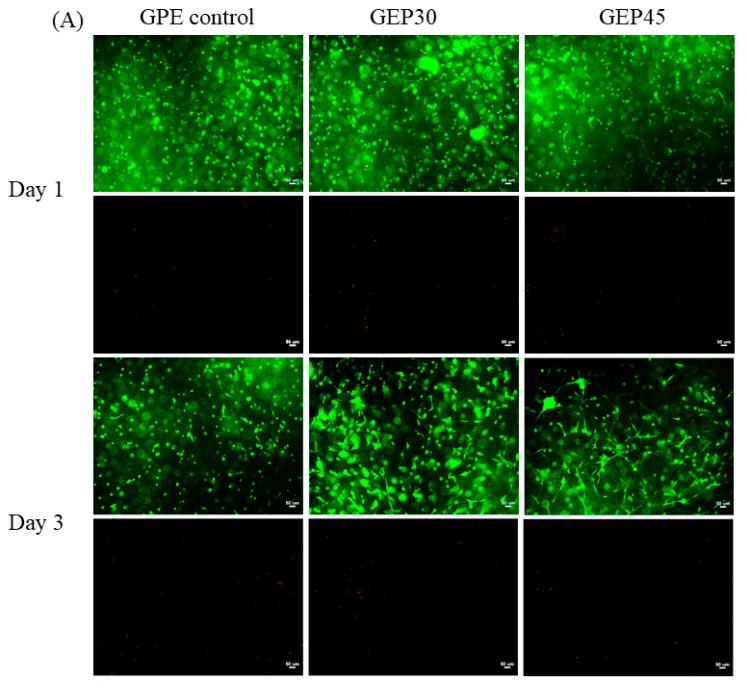

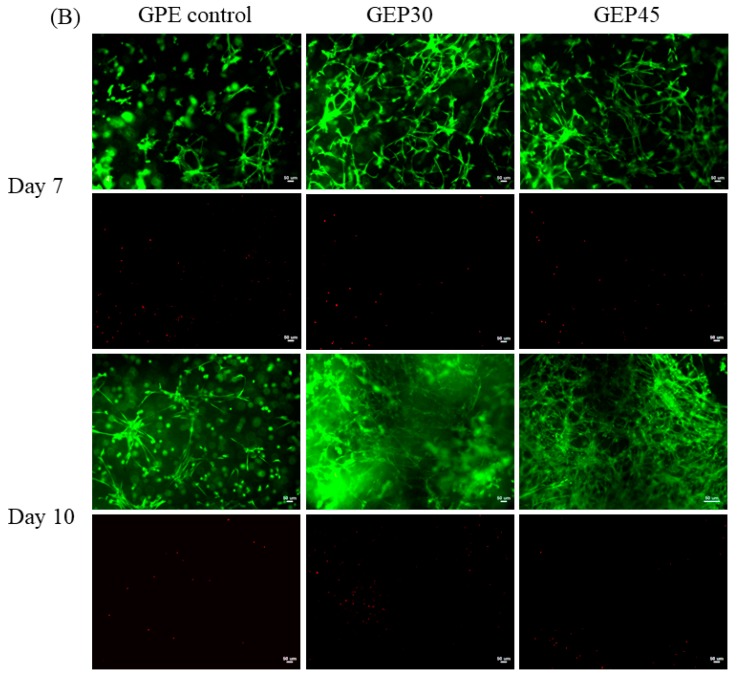
(**A**,**B**). Cell morphology in gelatin and elastin hybrid PEG hydrogels at d1, 3 were presented in (**A**), while that of day 7 and 10 showed in (**B**), respectively. The first row of each time point is the live cell-staining (green color, Calcein AM), while the second row is the dead cell-staining (red, EthD-1). All the scale bars are 50 µm.

Human dermal fibroblasts encapsulated in covalently cross-linked gelatin and elastin hybrid PEG hydrogels (GEP45, GEP30, and GPE control) were studied by staining with Calcein-AM and EthD-1. As shown in Figure 5, some of the NHDFs encapsulated in GEP45 and GEP30 hydrogels displayed evidence of attachment interactions on day 1, while the attachment of cells encapsulated in the GPE control hydrogels was delayed by a further 2 days, occurring on day 3. Moreover, on day 3, the level of NHDF attachment in GEP45 and GEP30 hydrogels was considerably greater than that achieved by the control NHDFs. By day 7, the cells in GEP45 and GEP30 hydrogels displayed cytoplasmic expansion and connections with neighboring cells, while the cells in the GPE control hydrogel had much less spreading and limited cell connections.

All three studied hydrogel had similar shear modulus but different elastin content. This design allows us to isolate the influence of elastin alone. The elastin in the GEP hydrogels provides additional protein content for increased cell guidance. Indeed, GEP45, with greater elastin connect than GEP30, demonstrated improved better cell growth. Although elastin does not contain the classic RGD motif, it is rich in cell-interacting sequences. These peptides include the GXXPG motifs such as VGVAPG and GVAPGV, which are bioactive sequences associated with cell chemotaxis, attachment and proliferation [61,62,63]. The elastin/laminin receptor also referred to as the elastin binding protein (EBP) interacts with the solubilized elastin via GXXPG sequences, and is expressed by several cell types including fibroblasts [29,64].

In the three hydrogels studied here, significant differences were observed on day 10: NHDFs in gelatin and elastin hybrid PEG hydrogels (GEP45 and GEP30) showed extensive cell spreading and the formation of intercellular networks, whereas NHDFs encapsulated in GPE control with physically incorporated elastin showed limited spreading and intercellular networks. As demonstrated by the gel fraction studies, the elastin in GPE control leached out from the hydrogel during the swelling and replacing of cell culture medium, hence the cell adhesive peptides within elastin were lost. All the cells encapsulated into these three hydrogels conferred for high cell viability from day 1 to day 10.

**Figure 6:**
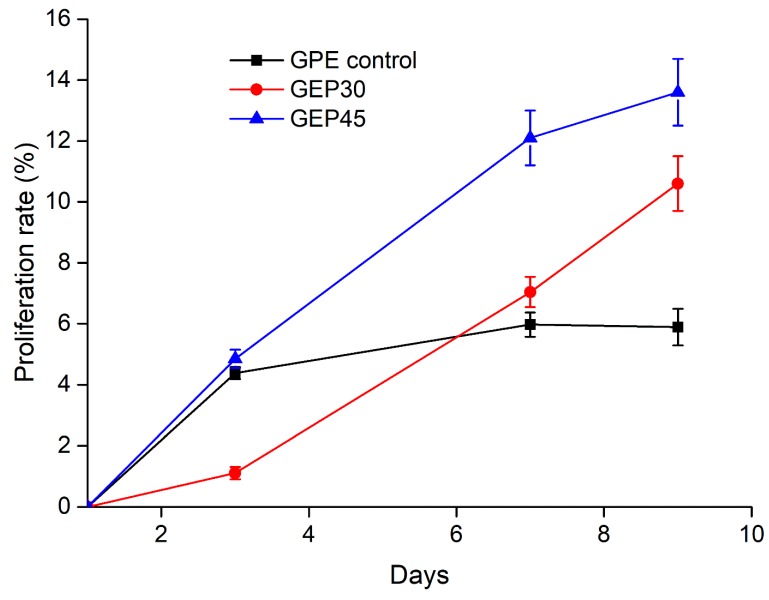
Cell proliferation analysis of NHDFs encapsulated in GEP45, GEP30 and GPE control at day 0, 3, 7 and 9. The single-parameter histograms of fluorescence EdU-488 levels for flow cytometry data was used to estimate the percentage of proliferation cells during 24 h EdU incubation. (“ Pro” and “Non-pro” represents the percentage of proliferative and nonproliferative cells respectively).

The proper mechanical property will help cells survival and promote the cell proliferation. For example, Haruka Oda et al. reported that the stem cells did not proliferate at the first three days when the G’ was above 1.1 kPa [51]. To further explore the interaction between cells and material, a cell proliferation study was conducted to investigate cell behavior on the three types of the hydrogel. The thymidine analog EdU incorporates into DNA over 24 h to label actively proliferating cells for flow cytometry [65]. The cells in the gelatin and elastin hybrid PEG hydrogel constructs displayed a greater rate of proliferation than those encapsulated in GPE control (Figure 6). Proliferating cells were detected by day 3 after they attached to the ECM protein and the hydrogels swelled to equilibrium (Figure 3B). Figure 4B presented that all the G’ of cell-laden hydrogels were below 1.1 kPa, thus the cells could start to proliferate after the encapsualtion. These results are in line with the cell morphology images reported in Figure 5. Over time the cells are able to expand their cytoplasm, elongate (Figure 5) and proliferate more in the hybrid hydrogels (Figure 6). In the case of the GPE control, this benefit is abrogated by the loss of elastin through leaching out of the elastin (lowest gel fraction and the highest swelling, shown in Section 3.2 and Figure 3B). In this work, we have shown clearly that covalently cross-linking soluble elastin into gelatin–PEG hydrogels confers a faster fibroblast proliferation rate for GEP45 (13.6%) and GEP30 (10.6%) compared to the GPE control (5.89%) at day 9. The mesh sizes are different and increase from GEP45 to GPE control, but that increase should favor proliferation [21,66]—we saw the reverse effect. We have tried to keep the moduli similar to avoid any purely mechanical effects on cell growth. Therefore, the observed cell proliferation must be attributed to the significant elastin content differences among the samples.

**Figure 7:**
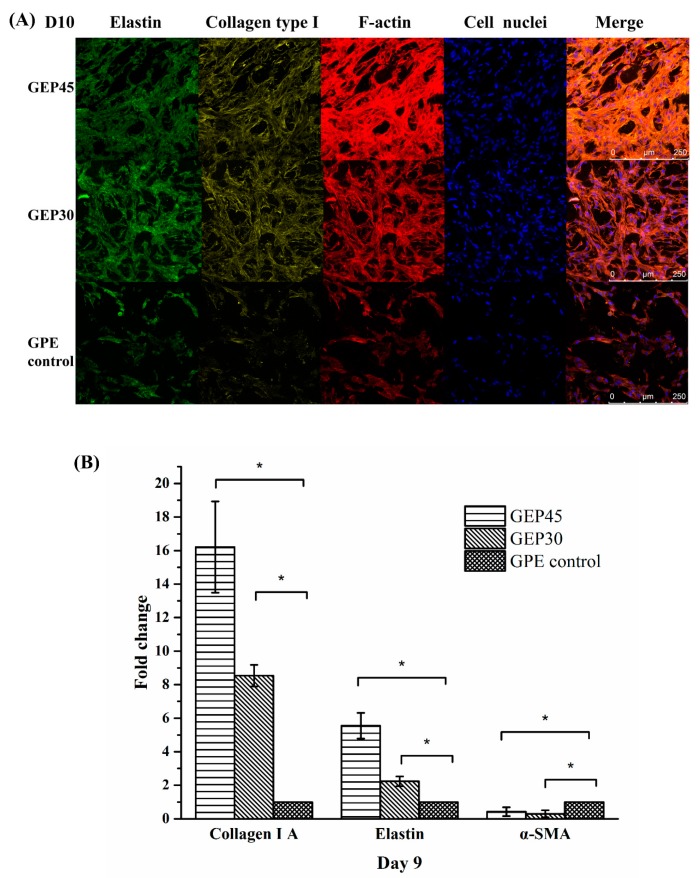
(**A**). Immunofluorescence staining for elastin (green), collagen type I (yellow), F-actin (red) and nucleic acid (blue) in GEP45, GEP30, and GPE control at day 10. Scale bar=250 µm. The antibodies of collagen type I and elastin only can be used for human source samples. The immunofluorescence staining images of cell-free hydrogel were showed in supporting file as the controls. (**B**) Effect of elastin addition on ECM proteins (COL1A1, ELN, and α-SMA) gene expression on day 9. Elastin addition resulted in a significant increase in collagen 1A1 and elastin at day 9. Gene expression was normalized to the housekeeping gene GAPDH and expressed as fold change versus NHDFs seeded GPE control hydrogels (considered as 1) at day 9. * denote *p* < 0.05. n = 4.

The matrix remodeling of the gelatin and elastin hybrid PEG hydrogels by the fibroblasts includes the production of structural proteins such as collagen and elastin. Suitable elastin regeneration is often an obstacle in tissue engineering, especially in dermal substitutes [67]. Elastic fibers are not readily resynthesized after injury due to difficulties in the expression of tropoelastin and related molecules, thus seriously hampering the quality and speed of healing. Therefore, burn survivors still suffer from excessive scarring and skin contractions, compromising recovery [22]. In order to further delineate the ECM deposition and cellular morphogenesis in the three hydrogels, NHDFs cultured within each hydrogel sample were fixed after 10 days culture, stained for elastin (green), type I A collagen (yellow), F-actin (red) and cell nuclei (blue) and finally imaged via confocal microscopy. As seen in Figure 7A, NHDFs within all samples displayed a certain level of F-actin bundling revealing the extent of the cytoskeleton. Cells in GEP45 and GEP30 showed significant cytoplasmic spreading and F-actin bunding compared with GPE control hydrogels. This indicated a more active interaction between fibroblasts and GEP45 or GEP30 because of the existence of covalently conjugated elastin. Higher elastin concentration in GEP45 provided more biological cues in the form of the GXXPG motifs [61], thus promoting the proliferation (Figure 6), while significant leaching of soluble elastin from the control decreased the chances for interactions. As we discussed in the introduction, the regeneration of elastin fibers is expected to take several years. Our results indicate, on the other hand, some regeneration of tropoelastin. Further research is needed to confirm the formation of elastin fiber.

Subsequent immunostaining analysis (Figure 7A) confirmed the presence of newly synthesized ECM proteins. Fibroblasts cultured within all three hydrogels were stained for collagen type I, elastin and F-actin. Newly synthesized elastin and collagen were more pronounced in GEP45 and GEP30 compared to GPE control; moreover, these proteins were more abundant around the fibroblasts. Collagen and elastin were considerably more noticeable within the GEP45 and GEP30 constructs than the control. The immunofluorenscence staining of cell-free hydrogels were presented in Figure S1. In addition, real-time PCR analysis (Figure 7B) quantitatively detected greater collagen 1A1 and elastin gene expression in the GEP45 hydrogel compared to the other two hydrogels, implying that the increased concentration of covalently conjugated elastin in the gelatin–PEG hydrogel promoted a higher rate of cell proliferation and ECM protein deposition. Willeke et al. demonstrated that the addition of solubilized elastin markedly improved the regeneration of collagen and elastin [29]. Aleksander et al. also have previously reported that dermal fibroblasts stimulated with a proteolytic digestion derivative of elastin, injected into the skin of nude mice or human skin explant model stimulated increased elastin gene expression as compared to untreated controls [68].

As mentioned above, the myofibroblast phenotype is implicated in fibrosis, and scar formation and wound contraction. As it is distinguishable from fibroblasts due to the cytoplasmic presence of α-SMA, the protein is a useful marker for the phenotype [69]. As shown in Figure 7B, α-SMA expression by NHDFs was reduced in both GEP45 (0.42 ± 0.25 fold less) and GEP30 (0.30 ± 0.21 fold less) as compared to the control sample (designated as 1). This indicates that the covalent conjugation of elastin into gelatin–PEG hydrogel did indeed suppress the fibroblast phenotype transdifferentiation to myofibroblast. Lamme et al., in their porcine excised wound model, collagen construct coated with 3% α-elastin and seeded with fibroblasts was used as a dermal matrix substitute combined with meshed split skin grafts [70]. This approach led to reduced wound contraction, improved tissue regeneration and absence of myofibroblasts compared to epidermal only transplantation. Matrix remodeling and elastin regeneration occurred both in the upper and lower dermis. In addition, Henry et al. also proved that elastin can suppress the transdifferentiation of phenotypically proliferative fibroblasts into contract myofibroblasts [59]. The molecular mechanism of this reaction has yet to be elucidated.

In summary, we have demonstrated that the incorporation of stable elastin as a component of a hybrid hydrogel has significant effects on fibroblast proliferation as well as on cellular phenotypical transformation. Specifically, elastin maintains the desired fibroblast form and reduces the presence of the deleterious myofibroblasts that are involved in wound contraction. Furthermore, the cross-linking mechanism is cytocompatible; hence the gel allows the suspension of cells without loss of viability, therefore creating an injectable hydrogel with the uniform cellular distribution.

## 4. Conclusions

Here we have characterized a novel hybrid gel for the cytocompatible entrapment and deposition of cells, with the additional properties of supporting cellular remodeling, and guiding the function and phenotype of encapsulated cells. Elastin has been shown to influence fibroblast behavior by promoting cell adhesion, morphological changes, and proliferation rate. It mediates gene expression at the level of transcription and protein secretion, leading to the expression of ECM gene products. Unlike most other biopolymers, the usefulness of elastin in a 3D gel is not purely in its matching of skin mechanical properties but also in its ability to convey instructions to seeded cells. Additionally, UV photopolymerization may generate reactive oxygen species (ROS) and reactive groups that impair cell physiology and genetic stability. Further study on cell functions after UV polymerization should be considered in the future experiments. In terms of dermal regenerative therapy, elastin may well prove to be a useful component of the next generation smart hydrogel with some ability to influence cell behavior favorably by minimizing scar tissue formation and enhancing healing.

## Figures and Tables

**Table 1:** The composition of GEP45, GEP30 and GPE control hydrogels. GPE control contains physically incorporated soluble elastin, gelatin–PEG precursor (4.80%(wt/v)) and 0.90%(wt/v) PEGDA (PEG diacrylate). Soluble elastin and PEGDA were covalently conjugated to the elastin–PEG precursor before fabricating GEP45 and GEP30, while PEGDA was added to GPE control to have similar mechanical properties as GEP45 and GEP30. The network cross-linking density was calculated from the rubber elastic theory. The mesh size was calculated based on the initial storage moduli and swelling ratio. (n = 3, *p* < 0.05).

Samples	Gelatin–PEG Concentration (wt/v %)	Elastin–PEG Concentration (wt/v %)	Elastin Concentration (wt/v %)	Elastin, as % of Solids	PEGDA Concentration (wt/v %)	Total Solid (wt/v %)	Network Cross-Linking Density (mol∙cm^−3^)	Initial Mesh Size (nm)
**GEP45**	4.80	4.50	0	27.1%	0	9.30	6.34 × 10^−5^ ± 0.67 × 10^−^^5^	22.4 ± 3.5
**GEP30**	4.80	3.00	0	21.5%	0	7.80	4.04 ×10^−5^ ± 0.58 × 10^−5^	30.3 ± 4.1
**GPE control**	4.80	0	1.50	23.8%- soluble, leaches out	0.90	7.20	2.15 ×10^−5^ ± 0.17 × 10^−^^5^	46.4 ± 4.2

**Table 2:** The constitution of gelatin–PEG and elastin–PEG precursor. The concentration of gelatin and elastin in precursor were measured by BCA method. The conjugation efficiency of amine groups and PEGDA was determined by trinitrobenzenesulfonic acid assay (TNBSA) method. (Mean ± STDEV, n = 3, *p* < 0.05).

Samples	Protein Ratio (%)	Protein Concentration (mg/mL)	PEG Ratio (%)	TNBSA (µg/mL)	Conjugation Efficiency (%)
Gelatin–PEG–acrylate	60.2 ± 4.5	32.5 ± 1.5	39.8 ± 4.5	160.3 ± 10.4	52.8 ± 6.5
Elastin–PEG–acrylate	56.4 ± 5.8	1.69 ± 1.7	43.6 ± 5.8	2.5 ± 0.1	59.6 ± 2.2

Note: Gelatin–PEG–acrylate solution after TFF purification and filtration with 0.22 µm membrane was stored in –70 °C for the cell encapsulation experiments as gelatin–PEG–acrylate was difficult to dissolve after lyophilization. The mass of gelatin–PEG–acrylate was achieved after lyophilization 1mL solution (with the subtraction of PBS salts: 16mg/mL). Elastin–PEG–acrylate was lyophilized after TFF purification. Elastin–PEG–acrylate was dissolved at 3 mg/mL for BCA and TNBSA measurement. Gelatin–PEG–acrylate was diluted 10 times before TNBSA assay. Original gelatin and elastin were used to prepare the standard curves of BCA assay, while lysine was used to prepare TNBSA standard curve. The TNBSA assay results of gelatin–PEG–acrylate and elastin–PEG–acrylate were normalized by the protein amount in the precursor for the calculation of conjugation efficiency.

**Table 3:** The viscosities of GEP 30, GEP 45 and GPE control precursor at different shear rates.

Samples	Viscosity (Pa∙s)		
	Shear Rate (s^−1^) 0.1	Shear Rate (s^−1^) 230	Shear Rate (s^−1^) 500
GPE control	0.0506	0.0133	0.0128
GEP30	648	0.510	0.2620
GEP45	1080	0.675	0.3450

## Supplementary Materials

The following are available online at https://www.mdpi.com/2073-4360/12/3/670/s1, Figure S1: Immunofluorescence staining for elastin (green) and collagen type I A (yellow) in cell free GEP45, GEP30 and GPE control, Figure S2: NHDFs were encapsulated into elastin-PEG hydrogel (no gelatin-PEG-acrylate or PEGDA).
