# Supplementary material for: Inclusion of Cross-Linked Elastin in Gelatin/PEG Hydrogels Favourably Influences Fibroblast Phenotype
Source: Polymers (Basel). 2020 Mar 17;12(3):670. doi: 10.3390/polym12030670 (PMC7183321; doi:10.3390/polym12030670)
Supplement: Supplementary file 1 [file polymers-12-00670-s001.pdf]

Collagen I A Elastin

Gelatin-PEG

GPE control

GEP 30

GEP 45

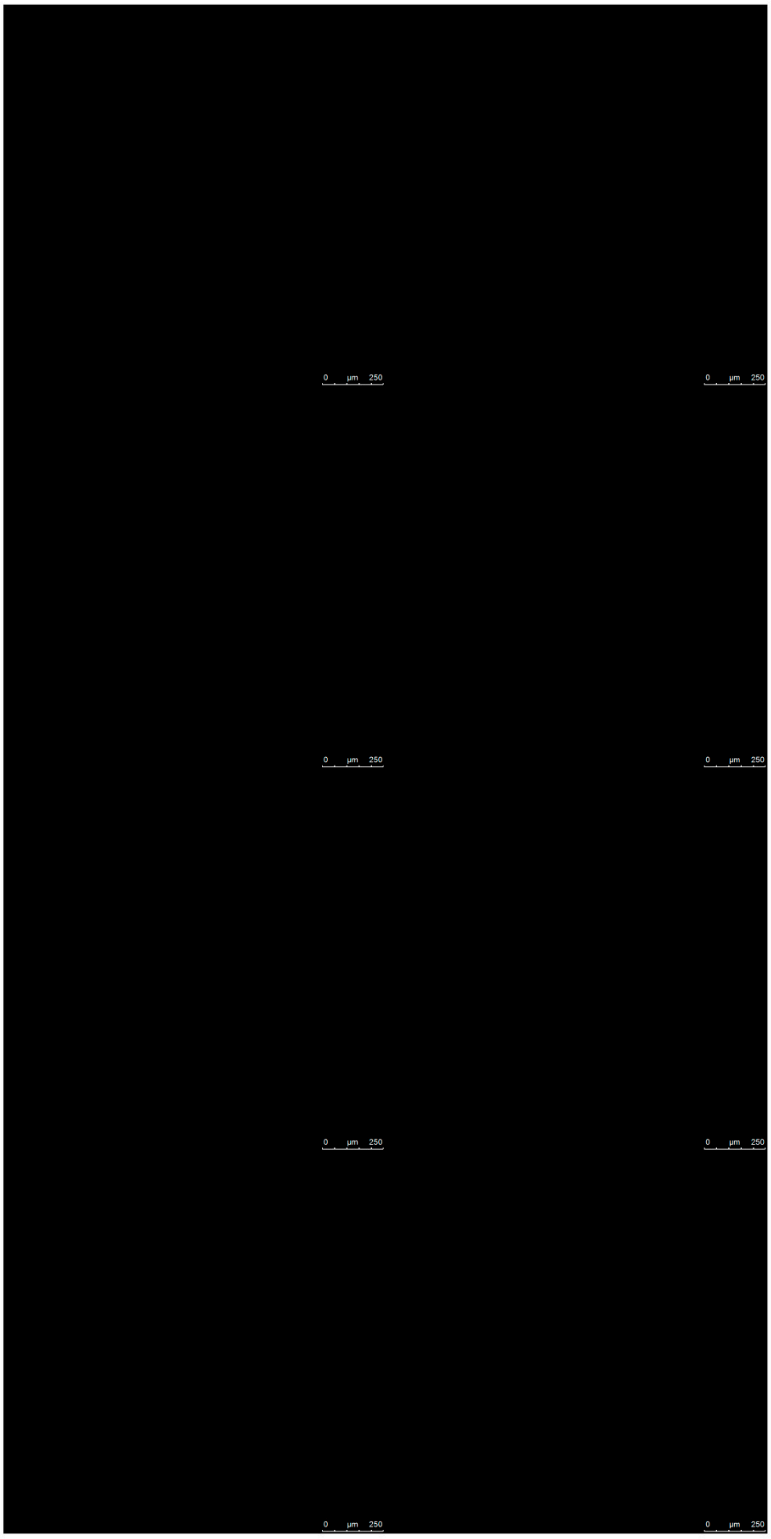

Figure S1. Immunofluorescence staining for elastin (green) and collagen type I A (yellow) in cell free GEP45, GEP30 and GPE control. Scale bar=250μm. Cell-free hydrogels have been stained the same as the cell encapsulated samples as negative controls. Above images proved that cell-free samples did not contribute to the staining results in Figure 7A.

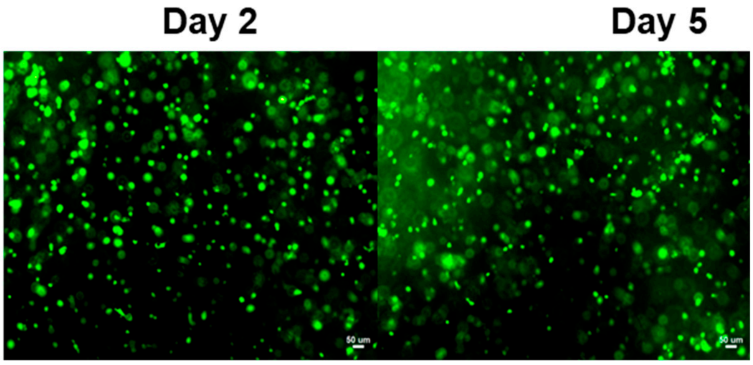

Figure S2. NHDFs were encapsulated into elastin-PEG hydrogel (no gelatin-PEG-acrylate or PEGDA). The cells were stained by Calcein AM at day 2 and day 5. Scale bar=50μm.
